# Safety and Efficacy of a Novel Centrifugal Pump and Driving Devices of the OASSIST ECMO System: A Preclinical Evaluation in the Ovine Model

**DOI:** 10.3389/fmed.2021.712205

**Published:** 2021-10-11

**Authors:** Sizhe Gao, Weining Wang, Jiachen Qi, Gang Liu, Jian Wang, Shujie Yan, Yuan Teng, Chun Zhou, Qian Wang, Weidong Yan, Qiaoni Zhang, Youjun Liu, Bin Gao, Bingyang Ji

**Affiliations:** ^1^Department of Cardiopulmonary Bypass, State Key Laboratory of Cardiovascular Disease, National Center for Cardiovascular Disease, Fuwai Hospital, Chinese Academy of Medical Science and Peking Union Medical College, Beijing, China; ^2^Faculty of Environment and Life, Beijing University of Technology, Beijing, China; ^3^Jiangsu STMed Technology Co. Ltd., Suzhou, China

**Keywords:** extracorporeal membrane oxygenation, centrifugal pump, critical care, ovine model, preclinical evaluation

## Abstract

**Background:** Extracorporeal membrane oxygenation (ECMO) provides cardiopulmonary support for critically ill patients. Portable ECMO devices can be applied in both in-hospital and out-of-hospital emergency conditions. We evaluated the safety and biocompatibility of a novel centrifugal pump and ECMO device of the OASSIST ECMO System (Jiangsu STMed Technologies Co., Suzhou, China) in a 168-h ovine ECMO model.

**Methods:** The portable OASSIST ECMO system consists of the control console, the pump drive, and the disposable centrifugal pump. Ten healthy sheep were used to evaluate the OASSIST ECMO system. Five were supported on veno-venous ECMO and five on veno-arterial ECMO, each for 168 h. The systemic anticoagulation was achieved by continuous heparin infusion to maintain the activated clotting time (ACT) between 220 and 250 s. The rotary speed was set at 3,200–3,500 rpm. The ECMO configurations and ACT were recorded every 6 hours (h). The free hemoglobin (fHb), complete blood count, and coagulation action test were monitored, at the 6th h and every 24 h after the initiation of the ECMO. The dissection of the pump head and oxygenator were conducted to explore thrombosis.

**Results:** Ten sheep successfully completed the study duration without device-related accidents. The pumps ran stably, and the ECMO flow ranged from 1.6 ± 0.1 to 2.0 ± 0.11 L/min in the V-V group, and from 1.8 ± 0.1 to 2.4 ± 0.14 L/min in the V-A group. The anticoagulation was well-performed. The ACT was maintained at 239.78 ± 36.31 s, no major bleeding or thrombosis was observed during the ECMO run or in the autopsy. 3/5 in the V-A group and 4/5 in the V-V group developed small thrombus in the bearing pedestal. No obvious thrombus formed in the oxygenator was observed. The hemolytic blood damage was not significant. The average fHb was 0.17 ± 0.12 g/L. Considering hemodilution, the hemoglobin, white blood cell, and platelets didn't reduce during the ECMO runs.

**Conclusions:** The OASSIST ECMO system shows satisfactory safety and biocompatibility for the 168-h preclinical evaluation in the ovine model. The OASSIST ECMO system is promising to be applied in clinical conditions in the future.

## Introduction

Extracorporeal membrane oxygenation (ECMO) has rescued many patients by providing pulmonary or cardiopulmonary support (1), especially during the COVID-19 pandemic (2). The ECMO circuit usually consists of a blood pump and its driver, an oxygenator, tubing and cannula, and several monitors (3). As a conventional ECMO system is composed of complex components, it is not always applicable on some special occasions, such as ECMO transportation and in-/out-of-hospital emergency conditions. Therefore, some portable devices were developed. Among those, Centrimag ECMO system (Levitronix LLC, MA, USA), Cardiohelp (Maquet Cardiopulmonary AG, Hirrlingen, Germany), Lifebox (Sorin, Milan, Italy), and Lifebridge B2T (Lifebridge Medizintechnik AG, Ampfing, Germany) were mostly used (4, 5). STMed has developed a novel portable ECMO system - the OASSIST ECMO System (Jiangsu STMed Technologies Co., Suzhou, China), consisting of a control console, a pump drive, and a single-use centrifugal pump. The pump was previously evaluated in hydraulic experiments, hemodynamic numerical simulations, and standard *in vitro* hemolysis experiments, showing good hydraulic performance and blood biocompatibility (6).

The purpose of this study was to evaluate the safety and biocompatibility of the OASSIST ECMO system in a 168-h ovine ECMO model.

## Materials and Methods

### Study Plan

All animal experiments were approved by the Institutional Animal Care and Use Committee (IACUC) of Fuwai Hospital [NO. 0101-2-20-HX(X)] and all procedures followed the NIH *Guide for the Care and Use of Laboratory Animals*. The experiment was completed at Beijing Key Laboratory of Pre-clinical Research and Evaluation for Cardiovascular Implant Materials, Animal Experimental Center of Fuwai Hospital (registration number: CNAS LA0009). All animals were subjected to routine quarantine and clinical examination before the experiment.

Ten healthy male Small Tailed Han sheep (Beijing Jinyutongfeng Trading Co., LTD, Beijing, China), 12–24 months old, were included in the study. All sheep had surgical implantation of the OASSIST ECMO system (Jiangsu STMed Technologies Co., Suzhou, China). Five underwent V-V ECMO (V-V group, *N* = 5) and five underwent V-A ECMO (V-A group, *N* = 5). The sheep were supported on ECMO for 168 h.

### The OASSIST ECMO System

**Figure 1:**
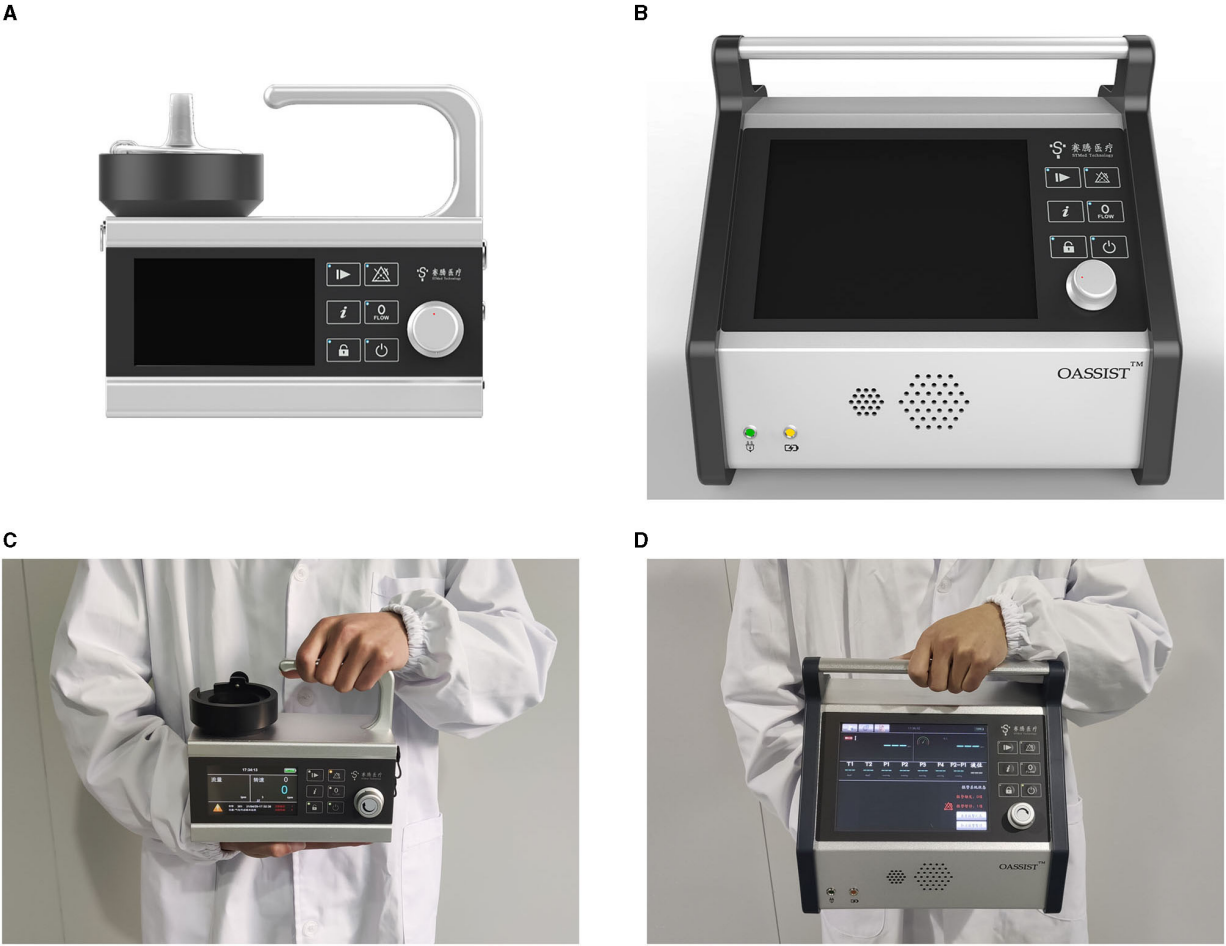
Macro-inspection the OASSIST ECMO system. **(A)** The macro-inspection of the pump drive. **(B)** The macro-inspection of the control console. **(C)** The pump drive hold by a staff. **(D)** The control console hold by a stuff.

**Figure 2:**
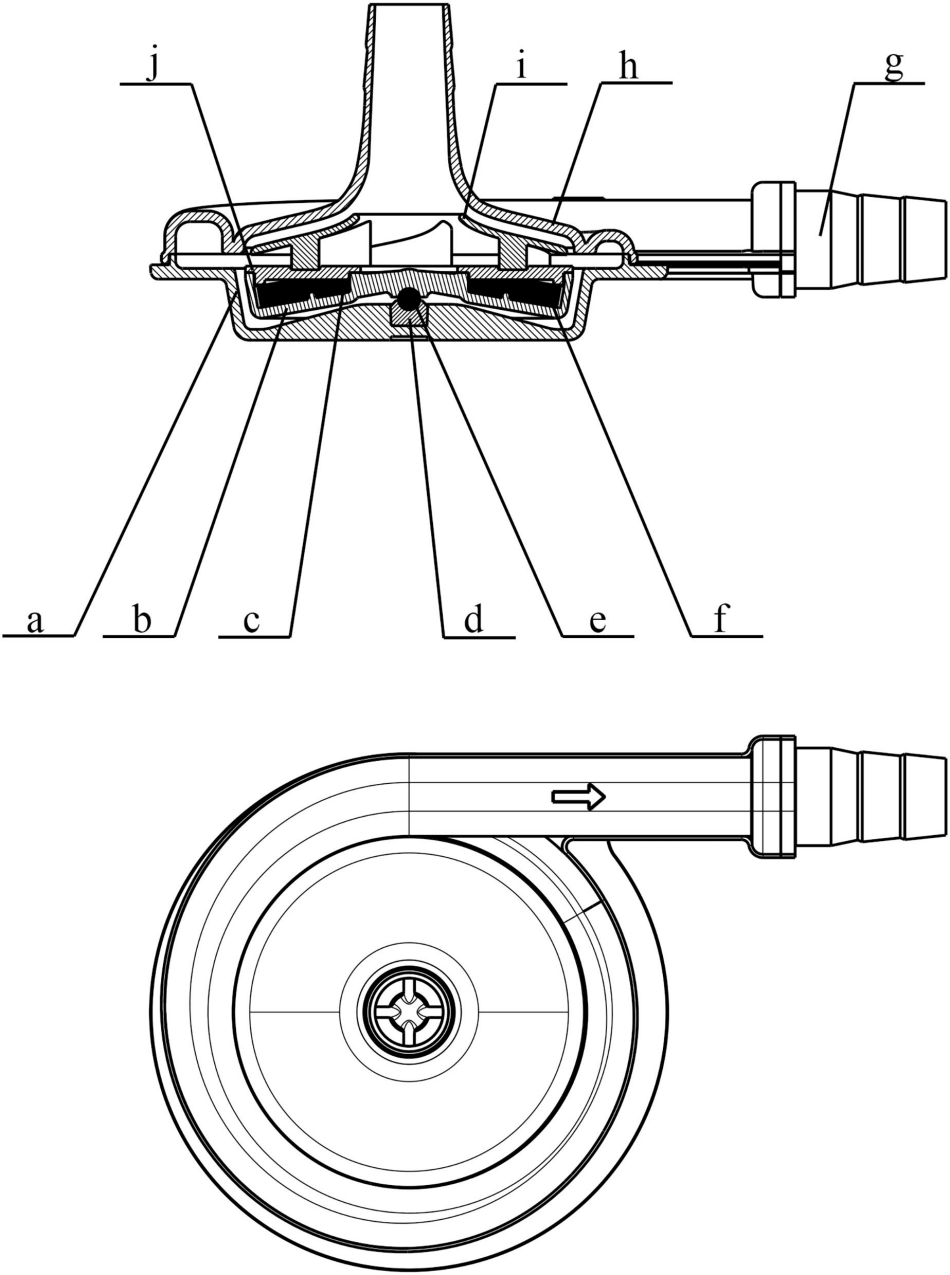
Schematic view of the pump head (STM CP-24 I) of the OASSIST ECMO system. a. the lower case of the volute; b. rotor; c. supporting ring; d. bearing pedestal; e. bearing; f. internal magnet; g. connector; h. the upper case of the volute; i. impeller; j. cap.

The OASSIST ECMO system consists of three components: the control console, the pump drive (OASSIST STM001), and the disposable centrifugal pump (STM CP-24 I) (Figure 1). The size of the control console is 290 × 260 × 210 mm, and the size of the pump drive is 200 × 190 × 110 mm. The control console sets pump speed and monitors operating parameters. The pump drive offers redundant direct control of the pump speed and flow/bubble detection, the battery of which could support for at least 180 min when alternating-current supply was interrupted. The centrifugal pump (Figure 2), driven by magnetic coupling, has a priming volume of 24 ml. The rotor of the pump has a contentious intersection area design to improve hydrodynamic efficiency, with a diameter of 45 mm. The rotating speed of the pump rates 1,000–5,500 rpm, and the optimal flow rate ranges from 3.0 to 5.0 L/min with a pressure head from 300 to 500 mmHg. And the pump could achieve a maximum flow rate of 8 L/min. The pump is designed to maximize its hydrodynamic efficiency and minimize shear stress.

### Oxygenator and Cannula

Commercial membrane oxygenators with hollow polymethyl pentene (PMP) fibers were used for both V-V and V-A ECMO. For V-V ECMO, the oxygenator kit was Hilite7000LT (XENIOS, Heilbronn, Germany); the 23 Fr Avalon Elite (Maquet, Rastatt, Germany) double-lumen cannula (DLC) was used. For V-A ECMO, the oxygenator kit was Hilite7000LT (XENIOS, Heilbronn, Germany) and BE-PLS 2050 (Maquet, Rastatt, Germany); the 18 Fr arterial cannula (Edwards Lifesciences, Irvine, CA, USA) and 24 Fr venous cannula (Edwards Lifesciences, Irvine, CA, USA) were used.

### Surgical Procedure

Anesthesia was induced with propofol (3–5 mg/kg) and maintained by isoflurane inhalation (2–3%) via mechanical ventilation and propofol injection (8–10 mg/kg/h). The right jugular vein of the V-V group; the right jugular vein and artery of the V-A group were exposed. A single-lumen central venous catheter was placed in the left jugular artery (arterial line), and a three-lumen central venous catheter was placed in the left jugular vein (venous line). Then, the initial systemic anticoagulation was induced by 120 IU/kg heparin. The target activated clotting time (ACT) of cannulation was higher than 250 s. The DLC was inserted under transthoracic echocardiography through the right internal jugular vein, with the tip positioned in the inferior vena cava. The arterial cannula was inserted through the right internal jugular artery, with the cannula descending 10–15 cm, while the venous cannula was inserted through the right jugular vein to the right atrium. Then, the pre-primed centrifugal pump head and the oxygenator were connected to the cannula. The animals were extubated immediately when consciousness returned.

### Measurement of the OASSIST ECMO System

The pre-set rotational speed was 3200-3500 rpm. An ultrasonic flowmeter (FBS 3/8” × 3/32”) was located between the pump outlet and the oxygenator. Three pressure probes were located before the pump inlet (pre-pump pressure), between the pump outlet and the oxygenator (post-pump pressure), after the oxygenator (post-oxygenator pressure), respectively. The adaptive temperature probe can be inserted into the oxygenator.

### Postoperative Care

**Figure 3:**
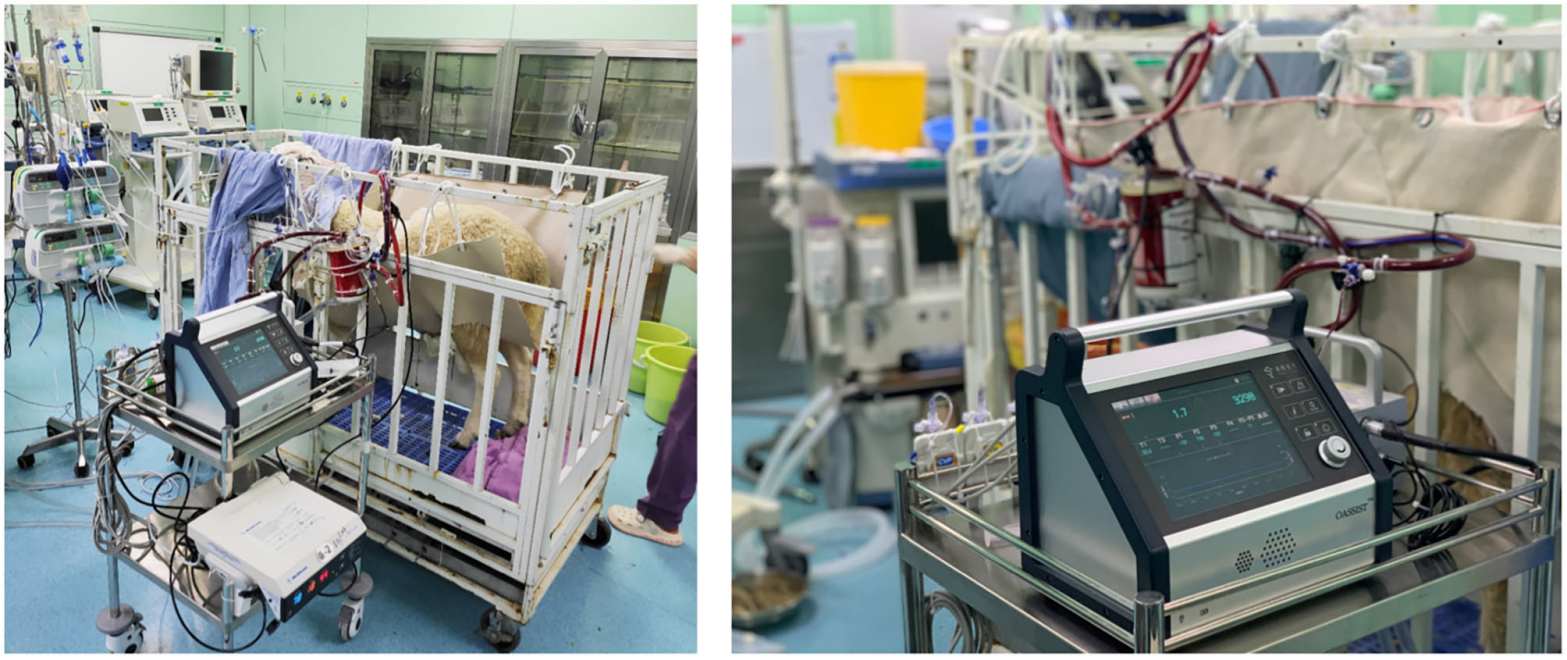
The sheep stayed conscious. The oxygenators were fixed to the cages, while the control console and the pump drive were placed on a cart.

The scheduled experiment duration was 168 h (h). The sheep were kept in the cage, conscious and feeding independently. A linen with four holes to put four legs in. And movement of the sheep's neck was restricted by another clause. The oxygenator was fixed to the cage, while the control console and the pump drive were placed on a cart (Figure 3). The heater-cooler was set at 38.5°C to maintain the normal body temperature of the sheep. In the first 24 h, flurbiprofen axetil (1–2 mg/kg) and dexmedetomidine (0.2–0.3 ug/kg•h) were administered intravenously. No sedation was needed after 24 h. Heparin was infused continuously to maintain ACT between 220 and 250 s. The initial heparin dose was 4–16 U/kg•h, and it was adjusted according to the ACT. Hemodynamic monitoring, intravenous fluids, drug injection, and blood sampling were conducted from the arterial and venous lines. After ECMO was weaned as scheduled, all sheep were euthanized by venous administration of potassium chloride (100 mg/kg) under the sedation of propofol (20 mg/kg).

### Biocompatibility Measurements

The vital signs, ECMO parameters (including speed, flow rate, pre-pump pressure, post-pump pressure, post-oxygenator pressure), and ACT (Hemochron Signature Elite, Hemochron, MA, USA) were recorded every 6 h. The free hemoglobin (fHb) (DiaSpect T Low Hemoglobin Analyzer, DiaSpect Medical GmbH, Sailauf, Germany), complete blood count (ADVIA 2120i, Siemens Healthcare, Erlangen, Germany), and coagulation action test (Fully Automated Coagulation Analyzer SF-8050, Beijing Succeeder Technology Inc, Beijing) were monitored at the 6th h and every 24 h after the initiation of the ECMO. After 168 h, the sheep were sacrificed, and autopsies of major organs were performed to explore internal embolism, thrombosis, or bleeding. After washing with 0.9% saline solution, the pump head, and oxygenator were dissected to explore the thrombosis.

### Statistical Analysis

All values were expressed as mean ± SD. Shapiro–Wilk test was used to test the normality of continuous variables. Normally distributed continuous variables were compared by Student's *t*-test and pairwise *t*-test, and non-normally distributed continuous variables were compared by the Mann–Whitney U-test or Wilcoxon test. All statistical testing was two-sided, and a *p*-value < 0.05 was considered significant. All statistical analyses were performed using GraphPad Prism 8 (GraphPad Prism, RRID:SCR_002798) and SPSS Version 26.0 (IBM SPSS Statistics, RRID:SCR_019096).

## Results

### Overall Performance of the OASSIST ECMO System

Ten sheep successfully completed the study duration without device-related accidents. One oxygenator and pump in the V-V group were changed at the 28th h after the initiation of the ECMO, due to the coagulation following the primary thrombosis formed in the cannulation site during the surgical procedure.

**Figure 4:**
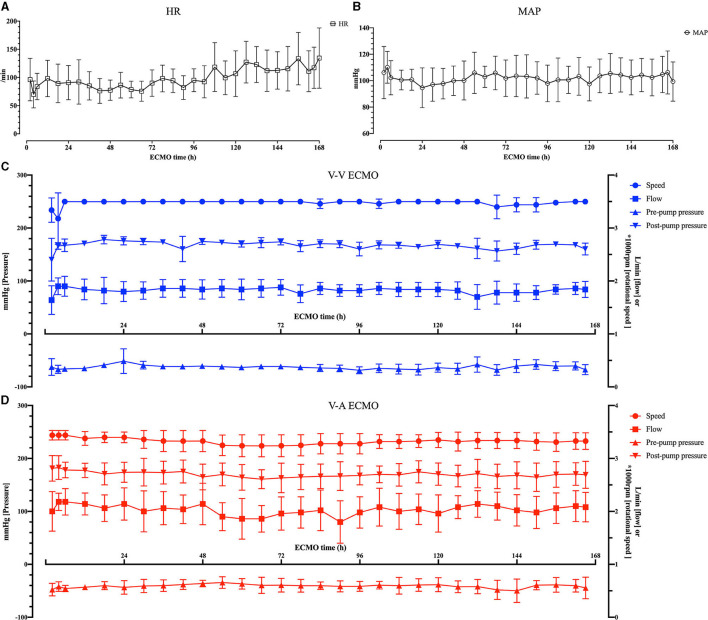
The vital signs of the sheep and the ECMO configurations. **(A)** The heart rate (HR) of the sheep. **(B)** The mean arterial pressure (MAP) of the sheep. **(C)** The rotational speed, flow, pre-pump pressure and post-pump pressure of the OASSIST ECMO system in the V-V group. **(D)** The rotational speed, flow, pre-pump pressure and post-pump pressure of the OASSIST ECMO system in the V-A group.

The pumps ran stably, the ECMO flow ranged from 1.6 ± 0.1 to 2.0 ± 0.11 L/min in the V-V group, from 1.8 ± 0.1 to 2.4 ± 0.14 L/min in the V-A group. The average pre-pump pressure was −52 ± 14.81 mmHg, and the average post-pump pressure was 169 ± 17.67 mmHg during the ECMO run (Table 1; Figure 4).

**Table 1:** Summary of ten sheep.

**No**.	**Configuration**	**Weight (kg)**	**Duration (h)**	**Termination**	**Rotational speed (rpm)**	**Flow (L/min)**	**Pre-pump pressure (mmHg)**	**Post-pump pressure (mmHg)**	**Transmembrane pressure (mmHg)**
VV1	V-V	56	168	Scheduled	3,497 ± 1	1.9 ± 0.03	−66.45 ± 7.06	170.03 ± 5.9	26.58 ± 1.15
VV2	V-V	60	168	Scheduled	3,498 ± 105.08	2 ± 0.11	−60.19 ± 6.12	164.68 ± 8.17	23.48 ± 3.21
VV3	V-V	63	168	Scheduled	3,498 ± 0.59	1.7 ± 0.07	−69.06 ± 6.22	172.55 ± 7.63	33.71 ± 8.16
VV4	V-V	59	168	Scheduled	3,499 ± 217.62	1.9 ± 0.14	−60.68 ± 5.56	162.94 ± 19.94	30.77 ± 5.81
VV5	V-V	60	196	Scheduled	3,497 ± 122.5	1.6 ± 0.1	−57.77 ± 12.41	162.77 ± 18.38	41 ± 11.99
VA1	V-A	58	168	Scheduled	3,499 ± 0.69	2.4 ± 0.14	−41.74 ± 12.78	197.23 ± 5.77	34.23 ± 4.08
VA2	V-A	54	168	Scheduled	3,198 ± 159.73	2 ± 0.29	−38 ± 9.14	166.48 ± 13.55	12.74 ± 1.48
VA3	V-A	55	168	Scheduled	3,501 ± 118.18	2.1 ± 0.22	−31.58 ± 6.32	178.52 ± 13.08	16 ± 2.7
VA4	V-A	56	168	Scheduled	3,248 ± 69.64	1.8 ± 0.14	−55.1 ± 5.69	162.06 ± 13.92	11.74 ± 2.5
VA5	V-A	57	168	Scheduled	3,198 ± 138.51	1.9 ± 0.24	−40.32 ± 6.13	149.06 ± 15.64	8.65 ± 2.24

### Coagulation Status

The anticoagulation was well-performed. The baseline ACT was 165.88 ± 16.88 s. After systemic anticoagulation, the ACT was maintained at 239.78 ± 36.31 s, and there was no difference between V-V and V-A group [241.67 ± 41.69 vs. 237.89 ± 30.35, *p* = 0.558 (Mann– Whitney U-test)].

**Figure 5:**
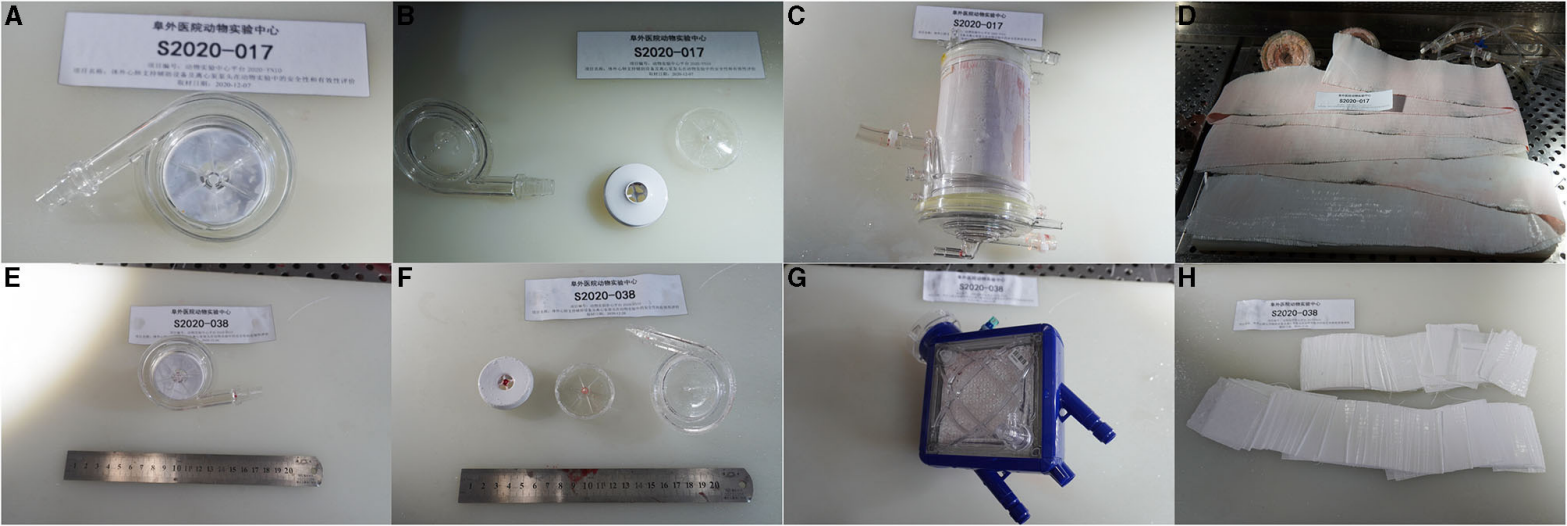
The representative picture of the pump head and oxygenator after 7-day ECMO. **(A)** A representative pump head that developed no obvious thrombus in the V-V group. **(B)** The dissection inspection of **(A)**. **(C)** The oxygenator that formed no obvious thrombus in the V-V group. **(D)** The membrane inside **(C)**. **(E)** A representative pump head that developed small thrombus in the bearing pedestal in the V-A group. **(F)** The dissection inspection of **(E)**. **(G)** The oxygenator that formed no obvious thrombus in the V-A group. **(H)** The membrane inside **(G)**.

No major bleeding or thrombosis was observed during the ECMO run or in the autopsy. Thrombosis around the cannulation site in the V-V group occurred more frequently compared to that in the V-A group (4/5 in the V-A group and 3/5 in the V-A group), but no vascular occlusion or stenosis was observed. 3/5 in the V-A group and 4/5 in the V-V group developed small thrombus in the bearing pedestal of the pump, and no obvious thrombus formed in the oxygenator was observed (Figure 5).

### Hemolytic Blood Damage Results

**Figure 6:**
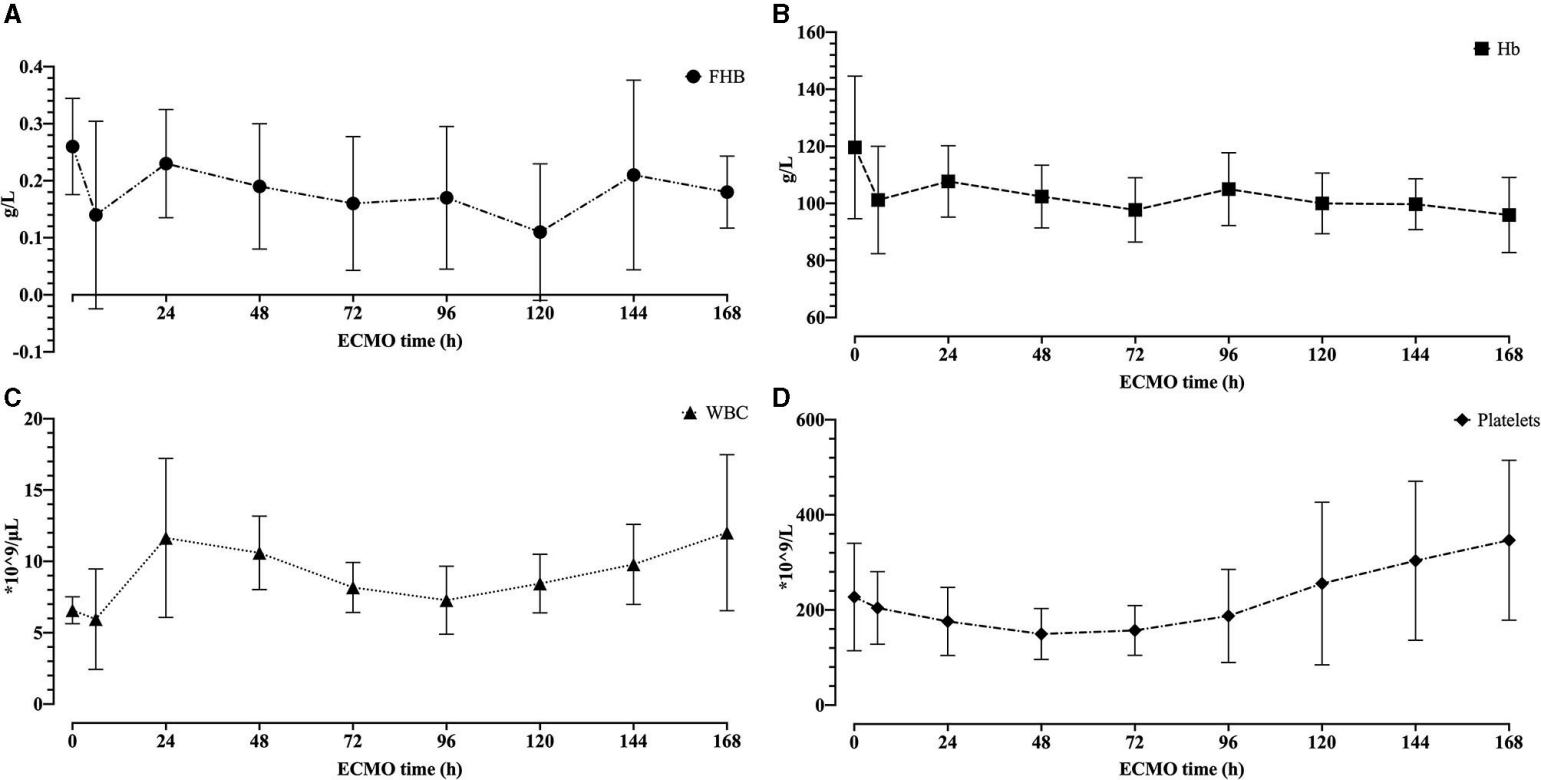
The hemolytic blood damage results. **(A)** The free hemoglobin for 7 days. **(B)** The hemoglobin for 7 days. **(C)** The white blood cell for 7 days. **(D)** The platelets for 7 days.

No sheep received blood product transfusion. The hemolytic blood damage was not significant. The fHb baseline was 0.26 ± 0.08 g/L. In a total of 80 tests of fHb after the initiation of ECMO, the average fHb was 0.17 ± 0.12 g/L. The fHb between the baseline and the 168th h was not statically different (0.26 ± 0.08 g/L vs. 0.18 ± 0.06 g/L, *p* = 0.066, Wilcoxon test). Considering hemodilution, we compared the hemoglobin, white blood cell (WBC), and platelets between the 6th h and the 168th h. No difference was observed in hemoglobin [101.2 ± 18.83 g/L vs. 95.9 ± 13.17 g/L, *p* = 0.536 (pairwise *t*-test)]. WBC and platelets elevated after 7-day ECMO [5.95 ± 3.52 × 10^3^/L vs. 12.01 ± 5.47 × 10^3^/L, *p* = 0.013 (Wilcoxon test); 204.3 ± 76.37 × 10^9^/L vs. 346.7 ± 168.13 × 10^9^/L, *p* = 0.022 (Wilcoxon test)]. Figure 6 shows how fHb, hemoglobin, WBC, and platelets varied.

## Discussion

This preclinical research shows that the centrifugal pump and ECMO device of the OASSIST ECMO system met safety and biocompatibility requirements satisfactorily, demonstrating three major results: First, the hemodynamic performance of the system was stable with no device-related accident (including pump stop, severe thrombosis, and severe hemolysis); Second, continuous heparin infusion provided sufficient anticoagulation, and no major bleeding or thrombosis was observed during ECMO run or in the autopsy; Third, blood damage revealed by fHb, RBC, WBC, and platelets was negligible.

In one experiment in the V-V group, the pump head and oxygenator had to be changed. Because during the cannulation attempt of the VV5 sheep, the cannula was punctured by the scalpel, and massive air was seen returning into venous line and centrifugal pump. The cannulation process was prolonged, and heparin was not supplemented in this process. During the first 28 h after the initiation of the ECMO, we found the transmembrane pressure increased precipitously (from 35 to 89 mmHg), indicating oxygenator thrombosis. Therefore, the occluded oxygenator and pump head were changed at the 28th hour. Long and narrow belt-shaped thrombus were found in both pump head and oxygenator, indicating it might be formed along the cannula and tubing near the punctuated site. Then, the pump ran for another 168 h.

Considering hemolytic blood damage, we tested fHb at baseline, the 6th h, and every 24 h after the initiation of ECMO. The fHb between the baseline and the 168th h was not statically different. However, the pre-ECMO fHb was slightly higher than every test after the ECMO run (Figure 6A), which may be caused by the different ways of blood sampling. A three-lumen central venous catheter was placed in the left jugular vein during the procedure, so the blood was collected through this venous line after the ECMO run. Comparatively, a syringe with a needle was used to draw blood at baseline. The needle may contribute to higher fHb.

### The OASSIST ECMO System Was Highly Portable

ECMO supports cardiac and respiratory failure and is frequently used as a bridge to transplantation, long-term mechanical circulatory support devices, and recovery, which has saved many critically ill patients. The OASSIST ECMO system is a compact heart-lung support system, composed of a single-use centrifugal pump, a pump drive, and a control console. The pump drive of the OASSIST ECMO system can be used independently with primed circuits in some emergency conditions, such as ECMO transportation and in-/out-of-hospital emergency treatment. The size of the pump drive is 200 × 190 × 110 mm and weighed 3 kg, which could be easily lifted and manipulated by any trained personnel. Since the system was much smaller than conventional ECMO devices, it could be applicable on more experimental and clinical occasions.

### The OASSIST ECMO System Provided Certain Hemodynamic Support

The pump head was optimized to support 3.0–5.0 L/min under the pressure of 300–500 mmHg. Fujiwara et al. evaluated a magnetically levitated, centrifugal blood pump on calves, the flow was around 3 L/min by central cannulation (7). Shankarraman et al. tested Levitronix^®^ Centrimag^®^ adult ECMO circuit on ovine acute pulmonary hypertension model, the average flow was 2.2 ± 0.1 L/min (8). Akiyama et al. also evaluated an ultra-compact durable ECMO system in sheep, and the flow rate ranged from 2.2 ± 0.7 L/min to 2.5 ± 0.1 L/min under the pump speed of higher than 4,000 rpm (9). In our experiment, the speed was set at 3,200–3,500 rpm. The ECMO flow ranged from 1.6 ± 0.1 L/min to 2.0 ± 0.11 L/min in the V-V group and from 1.8 ± 0.1 L/min to 2.4 ± 0.14 L/min in the V-A group (Table 1). We cannulated the V-V group by 23 Fr DLC because it was easier to fix and contributed to sheep's mobility. As is previously tested in the DLC evaluation sheep model, the 27 Fr DLC can provide around 2.0 L/min flow (10). Therefore, the OASSIST ECMO system can provide hemodynamic support that is comparable to previous animal studies. Although the JACC Scientific expert panel stated the flow of V-A ECMO should reach 4–6 L/min when supporting human patients (11), 2.0 L/min could satisfy hemodynamic needs in a healthy sheep model with a functional native heart.

### The OASSIST ECMO System Can Provide Durable Heart-Lung Support

STMed previously conducted *in vitro* durability tests (Supplementary Figure), the experiment system of which consisted of the control console, the disposable pump head driven by the pump drive, the tubing, and several monitors. Ten *in vitro* circuits (primed with 0.9% NaCl and glycerol) ran for 14 days, and no pump stop or other malfunction occurred. The durability tests were conducted under the supervision of National Institutes for Food and Drug Control. The OASSIST ECMO system can run for consecutive 14 days without any mechanical failure. Besides, when used independently without alternating-current supply, the pump drive can support for at least 180 min. The scheduled ECMO duration of previous preclinical evaluations ranged from several hours to at most 4 weeks (10, 12–14). Taken the previous samples and the durability of the PMP oxygenator together, we tested the single-use pump and ECMO device for 7 days in the ovine model. Thrombus formation remains a significant adverse event in mechanical circulatory support (MCS), including ECMO and ventricular assist devices (VADs) (15, 16). After the dissection of the pump and oxygenator, 7/10 pumps developed small thrombus in the bearing pedestal. But researchers didn't observe abnormal vibration or noise, intravascular hemolysis, or hemoglobinuria, which represented significant pump head thrombosis (17). Besides, no pump head was changed due to the primary pump thrombosis and the hemolytic blood damage was not significant. The combined results shows the system can run stably for at least 7 days.

### Limitations

Several limitations existed. First, the evaluation study didn't set a control group. To balance the preciseness with cost-effectiveness, the researchers designed a single-arm study. Second, the commercial PMP oxygenator was not the same between the V-V group and the V-A group due to availability, which may cause some bias when analyzing data. But on the other hand, this setting tested the compatibility of the OASSIST ECMO system with different oxygenators. Third, the animal model did not include a model of disease, as healthy sheep cannot completely be compared to patients with ARDS or cardiogenetic shock. By consulting experts on the animal experiment and researching previous studies, we found the success rate of disease model (such as acute lung injury and cardiogenic shock) was not controllable. Therefore, the healthy sheep model is more suitable for device evaluation.

## Conclusions

The OASSIST ECMO system shows satisfactory safety and biocompatibility during the 7-day preclinical evaluation in sheep. The OASSIST ECMO system could step to clinical evaluation and is promising to be a supportive device in critical and emergency medical conditions in the future.

## Data Availability Statement

The raw data supporting the conclusions of this article will be made available by the authors, without undue reservation.

## Ethics Statement

The animal study was reviewed and approved by the Institutional Animal Care and Use Committee (IACUC) of Fuwai Hospital.

## Author Contributions

YL, BG, BJ, and WW conceived and originally designed the research, then approved the final manuscript. SG and WW conducted the experiment and wrote the draft of the manuscript. SG analyzed the data. JQ, WY, and QZ extracted data from the electronic and paper databases. GL and SY conducted the experiment and revised the discussion section of the manuscript. JW, YT, CZ, and QW conducted the experiment and revised the materials and methods section of the manuscript. All authors contributed to the article and approved the submitted version.

## Conflict of Interest

WW reports support from Jiangsu STMed Technology Co. Ltd. Suzhou, China, which provides the OASSIST ECMO system for the experiment. The remaining authors declare that the research was conducted in the absence of any commercial or financial relationships that could be construed as a potential conflict of interest.

## Publisher's Note

All claims expressed in this article are solely those of the authors and do not necessarily represent those of their affiliated organizations, or those of the publisher, the editors and the reviewers. Any product that may be evaluated in this article, or claim that may be made by its manufacturer, is not guaranteed or endorsed by the publisher.
