# Supplementary material for: Safety and Efficacy of a Novel Centrifugal Pump and Driving Devices of the OASSIST ECMO System: A Preclinical Evaluation in the Ovine Model
Source: Front Med (Lausanne). 2021 Oct 11;8:712205. doi: 10.3389/fmed.2021.712205 (PMC8542924; doi:10.3389/fmed.2021.712205)
Supplement: Supplementary file 1 [file Data_Sheet_1.DOCX]

**Supplementary Figure.** The flow variation of the #1 *in vitro* circuits of the durability tests.
